# Supplementary material for: Splice-Junction-Based Mapping of Alternative Isoforms in the Human Proteome
Source: Cell Rep. Author manuscript; Available in PMC 2020 Jan 15. (PMC6961840; doi:10.1016/j.celrep.2019.11.026)
Supplement: 2 [file NIHMS1546469-supplement-2.zip › DF1/PXD010154/Liver/Liver_58_OGFOD3_GETHAILEAESIAYCK.pdf]

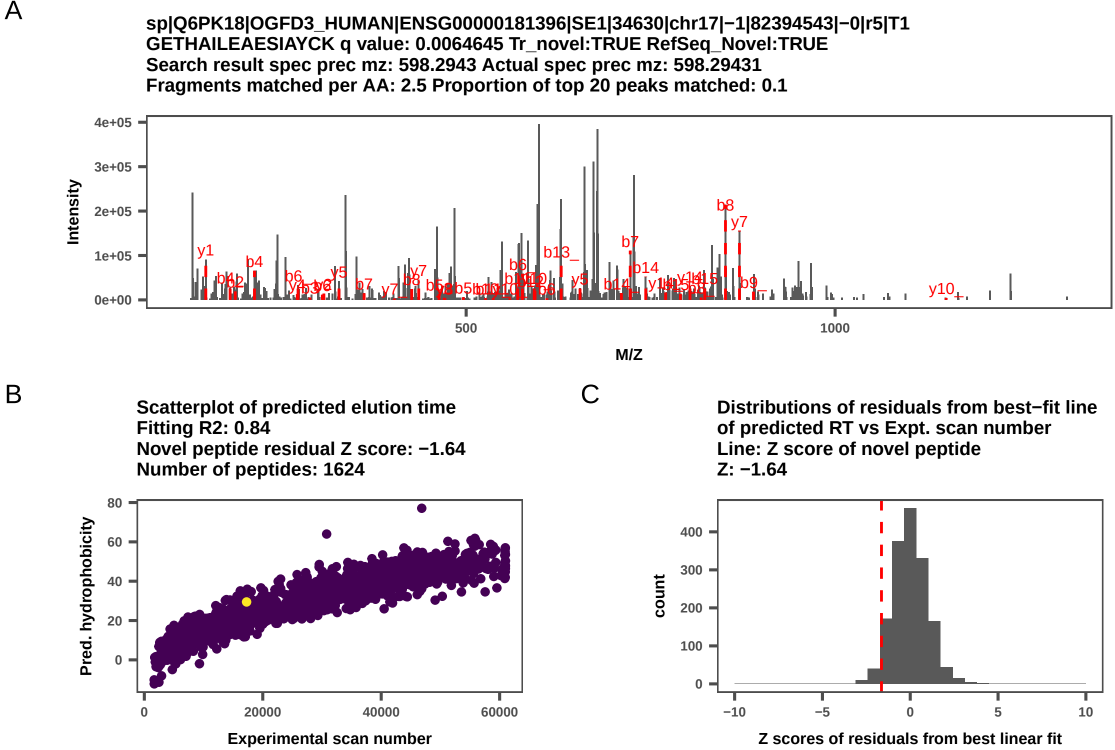
A
sp|Q6PK18|OGFD3_HUMAN|ENSG00000181396|SE1|34630|chr17|−1|82394543|−0|r5|T1
GETHAILEAESIAYCK q value: 0.0064645 Tr_novel:TRUE RefSeq_Novel:TRUE
Search result spec prec mz: 598.2943 Actual spec prec mz: 598.29431
Fragments matched per AA: 2.5 Proportion of top 20 peaks matched: 0.1
Intensity
4e+05
3e+05
2e+05
1e+05
0e+00
500
1000
M/Z
B
Scatterplot of predicted elution time
Fitting R2: 0.84
Novel peptide residual Z score: −1.64
Number of peptides: 1624
Pred. hydrophobicity
80
60
40
20
0
0
20000
40000
60000
Experimental scan number
C
Distributions of residuals from best−fit line
of predicted RT vs Expt. scan number
Line: Z score of novel peptide
Z: −1.64
count
400
300
200
100
0
−10
−5
0
5
10
Z scores of residuals from best linear fit
